# Intraoperative sampling for postoperative metagenomic next-generation sequencing to guide biofilm-targeted therapy for *Cutibacterium acnes* infective endocarditis complicated by ruptured sinus of Valsalva aneurysm: a case report

**DOI:** 10.3389/fcvm.2026.1707117

**Published:** 2026-03-05

**Authors:** Jie Liu, Ruijuan Wu

**Affiliations:** 1 Department of Critical Care Medicine, The Affiliated Qingyuan Hospital (Qingyuan People's Hospital), Guangzhou Medical University, Qingyuan, Guangdong, China; 2 Department of Intensive Care Unit, Hefei BOE Hospital Co., Ltd., Hefei, Anhui, China

**Keywords:** biofilm, *Cutibacterium acnes*, infective endocarditis, metagenomic next-generation sequencing, ruptured sinus of Valsalva aneurysms

## Abstract

**Background:**

*Cutibacterium acnes* is an easily overlooked pathogen in infective endocarditis (IE) due to its slow growth, propensity for biofilm formation, and high rate of culture-negative results. When complicated by structural heart disease such as a ruptured sinus of Valsalva aneurysm (RSVA), its indolent course can lead to severe hemodynamic compromise.

**Case summary:**

A 35-year-old male with a known ventricular septal defect (VSD) and unruptured aortic sinus aneurysm presented with persistent fever and progressive heart failure (NYHA class IV). Echocardiography revealed a ruptured right coronary sinus of Valsalva aneurysm (RCSVA) into the right ventricular outflow tract (RVOT) with a large vegetation. Blood cultures were negative. After 6 days of ineffective empirical antibiotic therapy, emergency surgery was performed to resect the aneurysm and vegetation and repair the cardiac structures. Intraoperatively, a vegetation sample was collected for metagenomic next-generation sequencing (mNGS). Postoperatively, mNGS identified *Cutibacterium acnes* with high sequence reads (1,284) and coverage (47.62%), enabling a definitive diagnosis. Pathology confirmed microcolonies and necrotic inflammation. The antibiotic regimen was switched to a regimen with potential activity against biofilms with oral doxycycline and intravenous clindamycin for 6 weeks. The patient's inflammatory markers normalized, and cardiac function recovered to NYHA class I, with no recurrence at 12-month follow-up.

**Conclusion:**

This case highlights the diagnostic synergy of intraoperative histopathology and mNGS for pathogen identification, underscores the rationale for biofilm-conscious adjuvant therapy, and reaffirms the crucial role of early surgical debridement and repair in achieving cure.

## Introduction

Infective endocarditis (IE) is a microbial infection of the endocardial surface of the heart, the diagnosis and treatment of which heavily rely on etiological evidence and precise eradication of the infectious focus (1). However, culture-negative IE accounts for approximately 10%–30% of all IE cases, often due to infection with fastidious organisms, biofilm formation, or prior antibiotic exposure (1, 2). *Cutibacterium acnes* (*C. acnes*), a cutaneous commensal, exemplifies a fastidious pathogen that frequently causes indolent IE. Its slow growth (often requiring anaerobic culture for ≥14 days) (3, 4), well-documented biofilm-forming capability, and low metabolic activity pose significant diagnostic challenges, often leading to misdiagnosis or delayed treatment (5). Structural heart diseases, such as ventricular septal defect (VSD) and sinus of Valsalva aneurysm, create endothelial turbulence and injury, predisposing to IE and creating a vicious cycle of infection and hemodynamic compromise (1).

Metagenomic next-generation sequencing (mNGS) technology, which enables unbiased detection of microbial nucleic acids in clinical samples, has significantly improved the diagnostic capacity for fastidious and biofilm-associated infections (6). However, its value in guiding perioperative decision-making, particularly in complex cases like ruptured sinus of Valsalva aneurysm (RSVA), and its role in informing biofilm-targeted therapy require further exploration. Furthermore, biofilm-mediated antibiotic tolerance mechanisms severely challenge traditional anti-infective regimens, necessitating optimized, molecular diagnosis-based individualized strategies (7).

This case report describes a patient with *C. acnes* IE complicated by RSVA and VSD. Following negative blood cultures and failed empirical therapy, the pathogen was identified via mNGS of an intraoperatively obtained tissue specimen, leading to an adjusted antibiotic regimen. This case highlights the role of molecular diagnostics combined with multidisciplinary collaboration in managing complex IE.

## Case description

A 35-year-old male was admitted on April 25, 2024, with a 2-month history of unexplained fever, peaking at 39.8 °C, accompanied by progressive fatigue and exertional dyspnea that had advanced to New York Heart Association (NYHA) class IV. The chronological sequence of clinical presentation, key interventions, and outcomes is summarized in Table 1. The patient had a known cardiac murmur since childhood, and prior echocardiography had suggested a VSD of approximately 5 mm and an unruptured aortic sinus aneurysm. Empirical antibiotic therapy administered at an outside hospital prior to admission had proven ineffective.

**Table 1:** Chronological timeline of patient presentation, management, and outcome.

Time point	Clinical event	Key investigations & interventions
∼2 months pre-admission	Onset of fever, fatigue	Outside hospital: empirical antibiotics (ineffective)
Admission (day 0)	NYHA IV heart failure, fever	Echo: RSVA, VSD, vegetation. Start Va. + Ceftriaxone
Day 1–6	Persistent fever	Blood cultures (x4 sets): negative. Clinical deterioration
Day 7	Emergency Surgery	Resection of RSVA/vegetation, patch repair, valvuloplasty
Intra-op		Sampling of vegetation for mNGS and culture
Postop day 2		mNGS result: *C. acnes* received
Postop day 2	Antibiotic Switch	Start Doxycycline + Clindamycin
Postop week 1	Defervescence, clinical improvement	Inflammatory markers declining (see Table 2)
Postop week 6	Completion of antibiotics	Inflammatory markers normalized
3-month follow-up	Asymptomatic	CT: resolved infiltrates. Echo: good repair
12-month follow-up	NYHA I, no recurrence	Echo: normal function

NYHA, New York Heart Association; Echo, echocardiography; RSVA, ruptured sinus of Valsalva aneurysm; VSD, ventricular septal defect; Va., vancomycin; mNGS, metagenomic next-generation sequencing; *C. acnes*, *Cutibacterium acnes*; CT, computed tomography.

**Figure 1:**
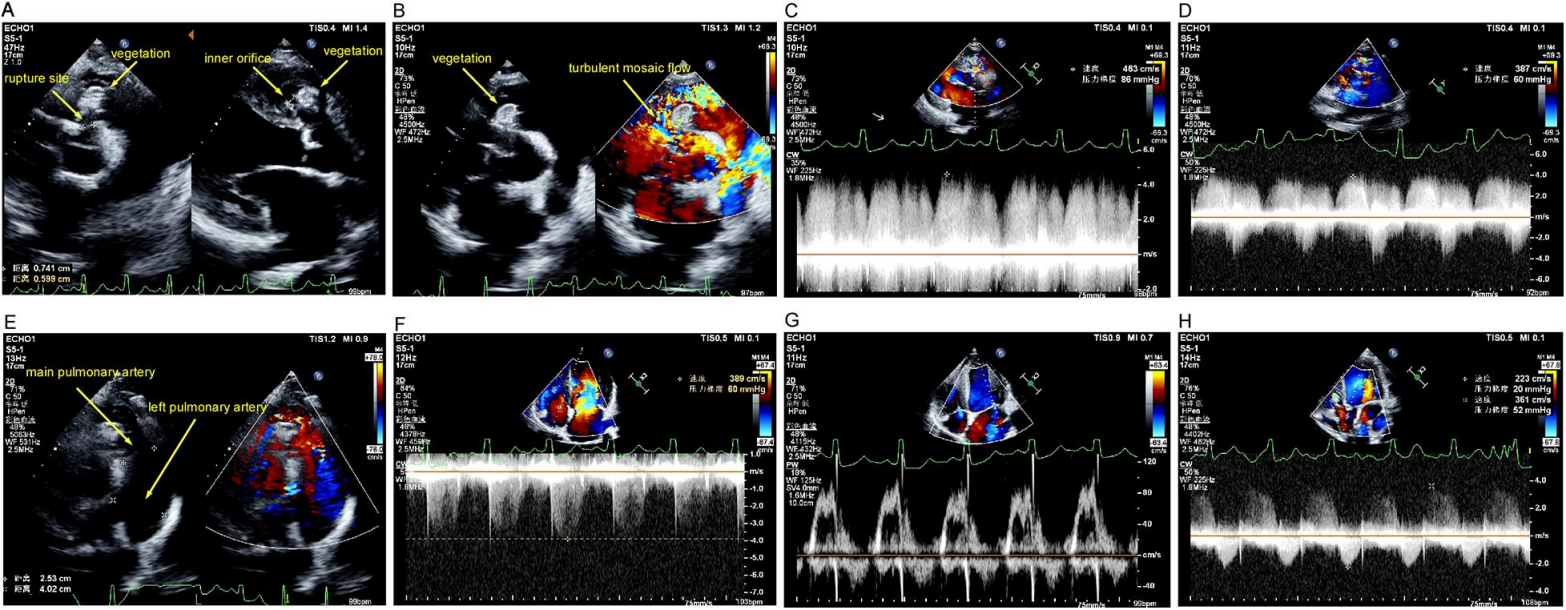
Preoperative transthoracic echocardiographic findings. **(A)** Parasternal short-axis and long-axis views showing a saccular outpouching of the right coronary sinus of Valsalva bulging into the infundibulum of the right ventricular outflow tract. The aneurysm wall appears echogenic, with a mass-like echo attached to its apex. A distinct rupture point is visible at the aneurysm neck; the inner orifice and rupture site measure approximately 0.60 and 0.74 cm in diameter, respectively. **(B)** Parasternal short-axis view with color Doppler demonstrating turbulent mosaic flow at the sinus of Valsalva aneurysm, within the right ventricular outflow tract, and into the pulmonary artery. **(C)** Parasternal short-axis view with continuous-wave Doppler detecting a biphasic shunt flow signal across the aneurysm rupture site, with a peak velocity of 463 cm/s and a pressure gradient of 86 mmHg. **(D)** Parasternal short-axis view with color Doppler showing turbulent mosaic shunt signals at the ventricular septum near the aneurysm base. Continuous-wave Doppler reveals a peak velocity of 387 cm/s and a pressure gradient of 60 mmHg. **(E)** Parasternal short-axis view revealing significant dilation of the main pulmonary artery (2.53 cm) and the left pulmonary artery (4.03 cm). **(F)** Apical four-chamber view with continuous-wave Doppler showing mild tricuspid regurgitation. The peak regurgitant velocity is 389 cm/s, estimating a tricuspid regurgitant pressure gradient (TRPG) of 60 mmHg. **(G)** Apical four-chamber view with color Doppler demonstrating mild to moderate mitral regurgitation. **(H)** Apical five-chamber view with color Doppler showing mild aortic regurgitation. The accompanying antegrade aortic flow is accelerated, with a maximum velocity of 223 cm/s, corresponding to a pressure gradient of 20 mmHg. RCSVA, right coronary sinus of Valsalva aneurysm; RVOT, right ventricular outflow tract; TRPG, tricuspid regurgitant pressure gradient; LVEDD, left ventricular end-diastolic dimension; LA, left atrial; RV, right ventricular; LVEF, left ventricular ejection fraction.

**Figure 2:**
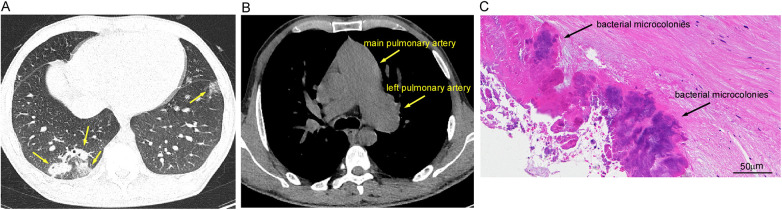
Preoperative chest CT and intraoperative histopathology. **(A)** Chest computed tomography (CT), lung window, demonstrating bilateral lower lobe pulmonary infiltrates (yellow arrows). **(B)** Chest CT, mediastinal window, revealing marked dilation and tortuosity of the main pulmonary artery (2.85 cm) and its left branch (4.03 cm) (yellow arrows). **(C)** Histopathological section of the resected vegetation (hematoxylin and eosin stain, 400× magnification) showing intense inflammatory infiltrates and bacterial microcolonies (black arrow). Scale bar: 50 µm. CT, computed tomography.

Upon presentation, the patient was febrile (39.2 °C) and tachycardic (110 bpm), and normotensive (100/65 mmHg). Physical examination revealed a continuous murmur and clinical signs of heart failure. Key laboratory findings at admission and their subsequent progression are summarized in Table 2. Transthoracic echocardiography revealed a complex pathophysiology. It confirmed a ruptured right coronary sinus of Valsalva aneurysm (RCSVA) protruding into the right ventricular outflow tract (RVOT), where the aneurysmal pouch caused significant infundibular obstruction. A large, irregular vegetation measuring 15 mm was attached to the aneurysm, and a biphasic shunt was present across the rupture site. Additionally, a separate subpulmonic VSD with left-to-right shunting was identified. The chronic volume and pressure overload resulted in severe dilation of the main pulmonary artery and its left branch. Secondary valvular pathologies included mild-moderate mitral regurgitation due to annular dilation, mild aortic regurgitation (likely related to cusp involvement by the aneurysm or jet lesion), and mild tricuspid regurgitation, which estimated a systolic pulmonary artery pressure of 60 mmHg (Figures 1A–H). Significant biventricular and left atrial enlargement was also noted, with measured dimensions as follows: left ventricular end-diastolic dimension 77 mm, left atrial diameter 56 mm, and right ventricular basal dimension 60 mm; left ventricular ejection fraction was preserved at 59% (data not shown). A chest computed tomography (CT) scan corroborated these findings, demonstrating bilateral pulmonary infiltrates and the markedly dilated, tortuous pulmonary arteries (Figures 2A,B).

**Table 2:** Key laboratory parameters at critical time points.

Parameter (normal range)	Admission	Pre-op (day 7)	Postop week 1	Postop week 6
WBC (3.5–9.5 × 10⁹/L)	10.23	12.59	7.8	6.2
Neutrophils (40%–75%)	85%	87.1%	65%	58%
Hb (130–175 g/L)	105.0	93.0	98.0	135.0
CRP (<8.0 mg/L)	32.66	91.3	7.4	2.1
PCT (<0.05 ng/mL)	2.3	6.1	0.04	0.01
ESR (<15 mm/h)	93	-	25	10
NT-proBNP (<125 pg/mL)	1,060	2,105	350	50

WBC, white blood cell count; Hb, hemoglobin; CRP, C-reactive protein; PCT, procalcitonin; ESR, erythrocyte sedimentation rate; NT-proBNP, N-terminal pro-brain natriuretic peptide.

In line with guideline recommendations, empirical intravenous antibiotic therapy was initiated with vancomycin and ceftriaxone. After 6 days of treatment, the patient remained febrile, and all four sets of blood cultures returned negative. Concurrently, laboratory markers indicated worsening inflammation and heart failure. Due to this clinical deterioration despite broad-spectrum therapy and the severe hemodynamic compromise from the structural lesions, a multidisciplinary heart team decided to proceed with emergency surgery on the seventh hospital day. Given the rapid clinical trajectory and the planned emergency surgery for definitive source control, the heart team opted for intraoperative tissue sampling as the highest-yield diagnostic step. Blood mNGS was not performed at presentation, as the sensitivity for detecting biofilm-associated pathogens like C. acnes under ongoing antibiotic therapy was considered uncertain (2), and the result would not have altered the immediate need for surgical intervention.

Intraoperative findings correlated precisely with the imaging, including a dilated main pulmonary artery, a ruptured RCSVA (Type I) communicating with the RVOT and bearing an approximately 15 mm vegetation, an 11 mm subpulmonic VSD, and a defect in the aortic right coronary cusp. The surgical procedure was tailored to address all components of the pathology: resection of the aneurysm and vegetation relieved the RVOT obstruction and achieved source control; reconstruction of the aortic sinus and closure of the VSD with bovine pericardial patches restored anatomical separation; concomitant aortic valvuloplasty and mitral annuloplasty addressed the valvular insufficiency; and pulmonary arterioplasty was performed to reduce the diameter of the chronically dilated pulmonary artery. The cross-clamp and cardiopulmonary bypass times were 98 and 135 min, respectively.

Histopathological examination of the resected tissue confirmed the presence of necrotic debris, intense inflammatory infiltrates, and bacterial microcolonies, consistent with an active infection (Figure 2C). A sample of the vegetation was collected intraoperatively under strict aseptic conditions for further analysis. The extraction and library preparation steps were performed once from a single tissue sample. For the mNGS analysis, DNA was extracted from approximately 50 mg of tissue using the QIAamp DNA Mini Kit (Qiagen, Hilden, Germany), and libraries were prepared with the Nextera XT DNA Library Prep Kit (Illumina, San Diego, CA, USA), following the manufacturers' protocols. Sequencing was performed on an Illumina NextSeq 550 platform. Bioinformatic analysis was performed using the Kraken2/Bracken pipeline, with the following quality control steps to minimize bias and contamination: raw sequencing reads were first processed with Trimmomatic (v0.39) (8) to remove adapters and low-quality bases (quality score <20); then, host-derived reads were depleted by aligning to the human reference genome (GRCh38) using Bowtie2 (v2.4.5) (9); finally, the remaining high-quality, non-host reads were classified taxonomically using Kraken2 (v2.1.2) (10) against a standard microbial database, and abundance was estimated with Bracken (v2.7) (11). A microorganism was deemed significant if it was absent from negative controls and had a high number of uniquely mapped reads with sufficient genome coverage. For bacterial pathogens, a breadth of coverage (the percentage of the reference genome covered by at least one sequencing read) greater than 30% has been suggested as a supportive threshold for clinical significance in mNGS-based diagnostics (12). mNGS of this sample definitively identified *C. acnes* with high confidence, reporting 1,284 reads and 47.62% breadth of coverage. No other microorganisms were detected above the reporting threshold, arguing against a polymicrobial infection or significant sample contamination. No antibiotic resistance genes were detected, and negative controls (including extraction and library preparation blanks) processed in parallel yielded no *C. acnes* reads, which strongly argues against laboratory contamination and validates the clinical significance of the result. In contrast, conventional aerobic and anaerobic cultures of the tissue specimen remained negative after a standard 5-day incubation period (prolonged incubation was not performed). The total turnaround time from sample collection to the final report was 72 h.

**Figure 3:**
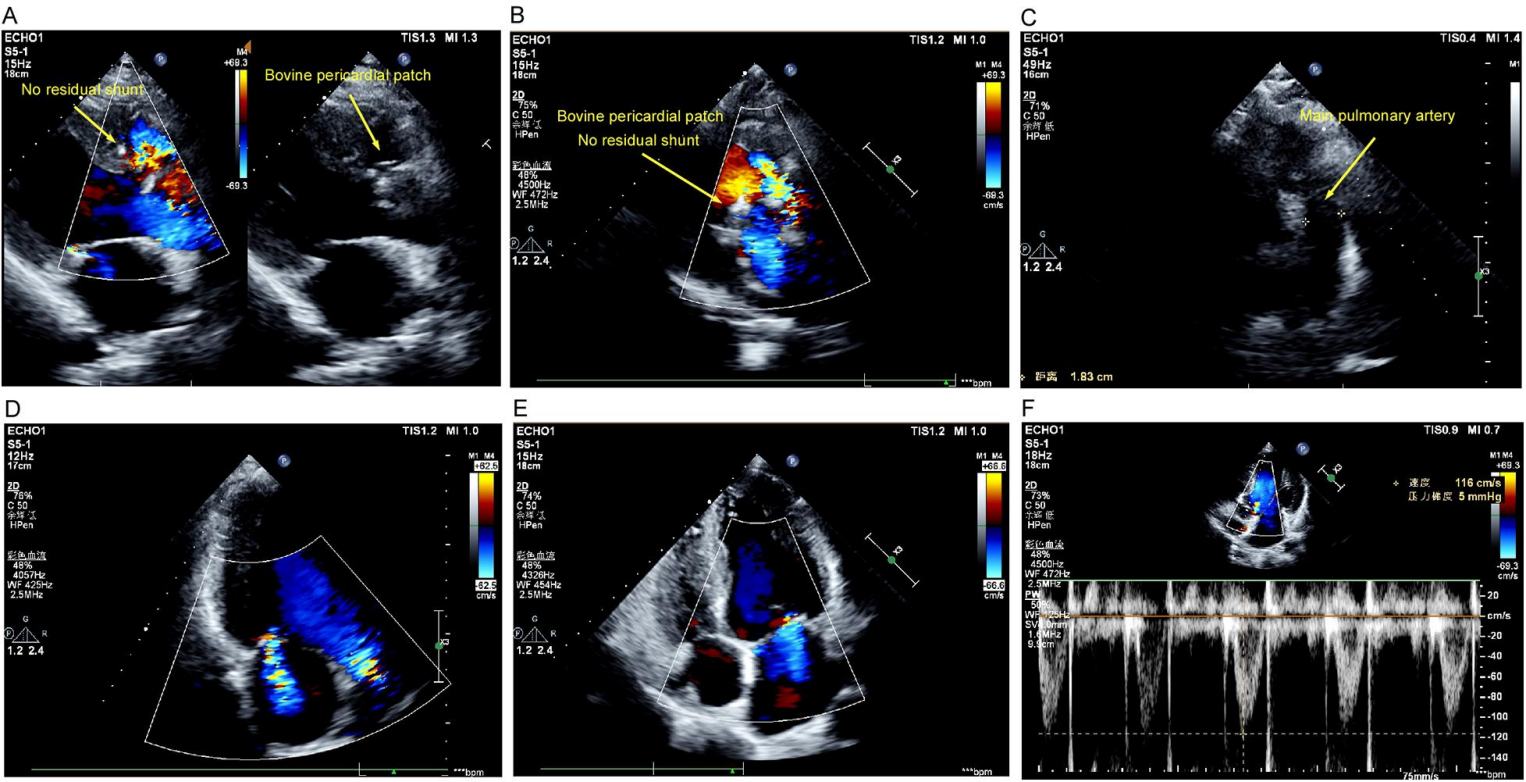
Postoperative transthoracic echocardiographic findings. **(A)** Parasternal long-axis view demonstrating a successful repair of the ruptured sinus of Valsalva aneurysm (RSVA) with a bovine pericardial patch at the right coronary sinus. The patch site and the adjacent right coronary cusp appear hyperechoic. No residual shunt is present. **(B)** Parasternal short-axis view showing a successful closure of the subpulmonic ventricular septal defect (VSD) with a bovine pericardial patch. The patch at the ventricular septum below the pulmonary valve appears hyperechoic and thickened, with preserved septal continuity and no residual shunt. **(C)** Parasternal short-axis view showing the main pulmonary artery, which has decreased in diameter to 1.83 cm postoperatively. **(D)** Apical four-chamber view with continuous-wave Doppler showing trivial tricuspid regurgitation. The peak regurgitant velocity is 180 cm/s, corresponding to a tricuspid regurgitant pressure gradient (TRPG) of 13 mmHg and an estimated systolic pulmonary artery pressure (SPAP) of 21 mmHg. **(E)** Apical four-chamber view with color Doppler demonstrating mild to moderate mitral regurgitation. **(F)** Apical five-chamber view with spectral Doppler showing mildly accelerated antegrade aortic flow (peak velocity 116 cm/s; pressure gradient 5 mmHg). Color Doppler revealed no significant aortic regurgitation. RSVA, ruptured sinus of Valsalva aneurysm; VSD, ventricular septal defect; TRPG, tricuspid regurgitant pressure gradient; SPAP, systolic pulmonary artery pressure.

**Figure 4:**
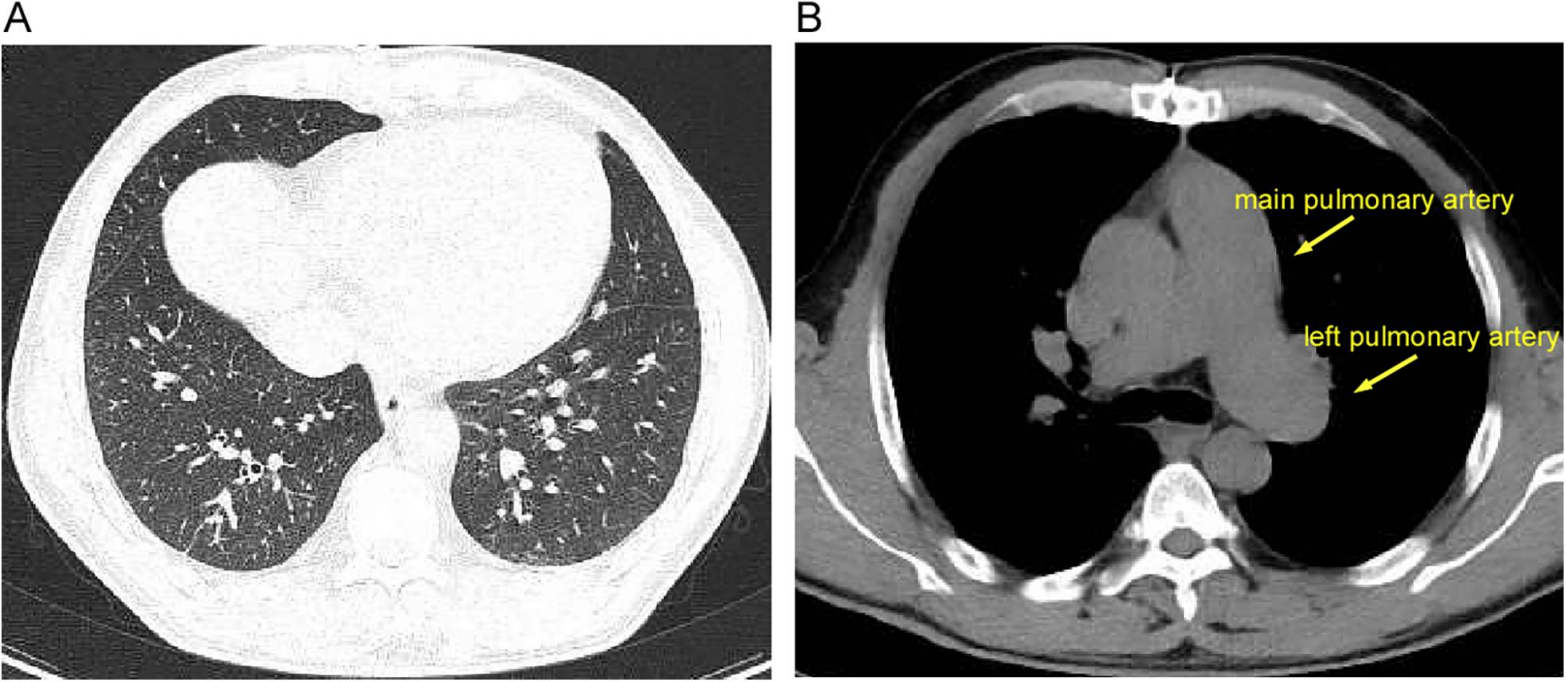
Postoperative chest CT at 3-month follow-up. **(A)** Chest CT, lung window, demonstrating resolution of the previously noted bilateral pulmonary infiltrates. **(B)** Chest CT, mediastinal window, showing reduced dimensions of the main pulmonary artery (1.83 cm) and its left branch (3.12 cm) (yellow arrows) compared to the preoperative study (see Figure 2B). CT, computed tomography.

The mNGS result was received on postoperative day 2, prompting a de-escalation of the antibiotic regimen to targeted therapy. This consisted of oral doxycycline and intravenous clindamycin, planned for a 6-week course. This combination was selected based on literature suggesting its potential efficacy against *C. acnes* biofilms and its penetration into slow-growing bacteria, alongside considerations regarding potential drug interactions with alternatives like rifampin in the immediate postoperative period. The patient defervesced within 48 h of initiating the new regimen. Inflammatory markers declined rapidly and normalized by the completion of antibiotic therapy. Follow-up echocardiography demonstrated successful anatomical repair with only mild residual valvular regurgitation, a normalized systolic pulmonary artery pressure (21 mmHg) (Figures 3A–F), and normalized chamber dimensions (not shown). A chest CT at 3 months confirmed the resolution of the pulmonary infiltrates and a reduction in pulmonary artery dimensions (Figures 4A,B). At the 12-month follow-up, the patient was asymptomatic (NYHA class I), with normal cardiac function and no evidence of infection recurrence. The importance of lifelong endocarditis prophylaxis was reinforced.

## Discussion

This case illustrates the successful application of intraoperative sampling for mNGS to guide the postoperative management of a rare and synergistic presentation of culture-negative IE: a ruptured sinus of Valsalva aneurysm with fistulization, complicated by destructive endocarditis from a fastidious, biofilm-prone pathogen. It underscores the diagnostic challenges in such scenarios and highlights a pragmatic, synergistic approach combining advanced molecular diagnostics, timely surgery, and targeted antimicrobial therapy.

The diagnostic limitations of traditional microbiology in *C. acnes* IE are well-documented (4, 5). In this context, mNGS of the intraoperatively acquired vegetation served as a decisive tool, providing a rapid and unbiased pathogen identification, a finding strongly supported by histology. This aligns with studies demonstrating the superior sensitivity of mNGS over culture in IE diagnosis (13, 14). However, the potential for contamination with skin flora like *C. acnes* necessitates rigorous protocols and interpretive caution. In our case, the high sequence count, histological correlation, and negative procedural controls strengthen the validity of the result. The sequence coverage of 47.62% for *C. acnes*, which surpasses suggested thresholds for clinical significance (12), provides strong additional support for its pathogenic role. The 72-hour turnaround time, while not intraoperative, was sufficiently rapid to guide early postoperative therapy adjustment.

The preoperative failure of broad-spectrum bactericidal agents (vancomycin and ceftriaxone), combined with the histopathological finding of bacterial microcolonies—a recognized tissue correlate of biofilm aggregates—strongly supports the inference of a biofilm-associated component to the infection (15–17). It is critical to emphasize that definitive cure was achieved through surgical debridement, which provided essential source control. The postoperative antibiotic regimen was adjuvantly selected based on this inferred biology and the pathogen identity to target potential residual, sheltered organisms, a rationale that directly informed our antimicrobial choice.

*C. acnes* biofilms, encased in an extracellular polymeric substance, significantly reduce antibiotic penetration and increase minimal inhibitory concentrations (17). Notably, doxycycline has demonstrated efficacy against *C. acnes* biofilms *in vitro* (15). While the exact mechanism requires further elucidation, it may be related to its anti-inflammatory properties and potential inhibition of matrix metalloproteinases that could contribute to biofilm integrity. Clindamycin, on the other hand, retains activity against slow-growing bacteria and can effectively penetrate bacterial cells (18). A definitive diagnosis of biofilm would require specialized techniques like electron microscopy, which was not performed here, representing a limitation of our report (17, 19). Furthermore, in the context of our therapeutic decision, the absence of detected resistance genes by mNGS was reassuring but not definitive. The inability to obtain a clinical isolate for susceptibility testing remains a significant gap, particularly given emerging clindamycin resistance among *C. acnes* (20). The fact that both blood and tissue cultures remained negative after standard incubation periods—likely insufficient for a slow-growing organism like *C. acnes*—further underscores the limitations of routine microbiological workflows in such scenarios. This underscores the importance of explicitly requesting prolonged anaerobic cultures in cases of suspected culture-negative IE or utilizing molecular methods like mNGS to bridge this diagnostic gap. The potential role of rifampin was considered, but it was deferred due to potential interactions with other medications in the immediate postoperative phase.

The decision for early surgical intervention was critical, addressing both source control and the underlying structural defects, as recommended by guidelines (1). The multidisciplinary collaboration was indispensable in this timely decision.

This report has several limitations. First, the diagnosis of biofilm involvement is inferred rather than confirmed by direct methods like electron microscopy. Second, the lack of a positive culture precluded susceptibility testing. Third, Gram staining was not performed, which could have provided complementary morphological evidence. Fourth, regarding mNGS, the analysis was performed on a single sample—a design that limits the ability to definitively rule out sample contamination, though no other microorganisms were detected. Additionally, mNGS detects DNA regardless of microbial viability; however, the histopathological evidence of active infection supports the clinical relevance of the *C. acnes* finding in this acute setting. Fifth, pulmonary artery dimensions were measured on non-contrast CT, which may affect absolute accuracy but not the reliability of the comparative reduction observed. Finally, as a single case report, its generalizability is limited.

## Conclusion

This case exemplifies a successful multidisciplinary approach to managing complex, culture-negative IE caused by a fastidious pathogen. The key takeaways are threefold: Firstly, intraoperative sampling for mNGS proved pivotal, enabling a definitive etiological diagnosis and guiding a targeted, biofilm-conscious therapeutic strategy where conventional methods failed. Secondly, the case underscores that low-virulence pathogens like *C. acnes* can cause devastating infections in the setting of structural heart disease, and a high index of suspicion for biofilm-associated tolerance is warranted. Finally, early surgical intervention for source control and anatomical correction remains the cornerstone of management in such complex cases. Efforts to optimize rapid molecular diagnostics and explore enhanced anti-biofilm strategies are imperative.

## Data Availability

The original contributions presented in the study are included in the article/Supplementary Material, further inquiries can be directed to the corresponding author.
